# Dyskeratosis congenita: natural history of the disease through the study of a cohort of patients diagnosed in childhood

**DOI:** 10.3389/fped.2023.1182476

**Published:** 2023-08-01

**Authors:** M. L. Uria-Oficialdegui, S. Navarro, L. Murillo-Sanjuan, C. Rodriguez-Vigil, M. I. Benitez-Carbante, C. Blazquez-Goñi, J. A. Salinas, C. Diaz-de-Heredia

**Affiliations:** ^1^ Pediatric Hematology and Oncology Division, Hospital Universitari Vall d´Hebron, Barcelona, Spain; ^2^ Pediatric Division, Hospital Universitario SonEspases, Palma de Mallorca, Spain; ^3^ Pediatric Oncohaematology Unit, Paediatric Division, Hospital Universitario Miguel Servet, Zaragoza, Spain; ^4^ Hematology Division, Hospital Virgen del Rocío, Sevilla, Spain

**Keywords:** dyskeratosis congenita, telomeres, inherent bone marrow failure, multisystem disease, aplastic anaemia, hematopoeietic stem cell transplantation

## Abstract

**Background:**

Dyskeratosis congenita (DC) is a multisystem and ultra-rare hereditary disease characterized by somatic involvement, bone marrow failure, and predisposition to cancer. The main objective of this study is to describe the natural history of DC through a cohort of patients diagnosed in childhood and followed up for a long period of time.

**Material and methods:**

Multicenter, retrospective, longitudinal study conducted in patients followed up to 24 years since being diagnosed in childhood (between 1998 and 2020).

**Results:**

Fourteen patients were diagnosed with DC between the ages of 3 and 17 years (median, 8.5 years). They all had hematologic manifestations at diagnosis, and nine developed mucocutaneous manifestations during the first decade of life. Seven presented severe DC variants. All developed non-hematologic manifestations during follow-up. Mutations were identified in 12 patients. Thirteen progressed to bone marrow failure at a median age of 8 years [range, 3–18 years], and eight received a hematopoietic stem cell transplant. Median follow-up time was 9 years [range, 2–24 years]. Six patients died, the median age was 13 years [range, 6–24 years]. As of November 2022, eight patients were still alive, with a median age of 18 years [range, 6–32 years]. None of them have developed myeloblastic syndrome or cancer.

**Conclusions:**

DC was associated with high morbidity and mortality in our series. Hematologic manifestations appeared early and consistently. Non-hematologic manifestations developed progressively. No patient developed cancer possibly due to their young age. Due to the complexity of the disease multidisciplinary follow-up and adequate transition to adult care are essential.

## Introduction

The natural history of dyskeratosis congenita (DC) (ORPHA:1775) is difficult to establish as the disease is very rare (1 case per 1,000,000 inhabitants) (1, 2) and has a wide range of clinical presentations. DC is a multisystemic syndrome characterized by mucocutaneous abnormalities (nail dystrophy, abnormal skin pigmentation and oral leukoplakia), bone marrow failure (BMF), and a predisposition to cancer (3–6). The pattern of inheritance is complex, as DC can be inherited in an autosomal dominant (AD), autosomal recessive (AR), or X-linked pattern, and *de novo* mutations have also been described (7–12). Mutations in 18 genes with an important role in telomere biology have been identified to date. These genes have a principal role in telomere maintenance and patients usually have very short telomeres (2). Diagnosis is challenging, as there are no clear genotype-phenotype correlations, and phenotypes can range from oligosymptomatic forms to severe disease (1, 2, 6, 13). In addition, there are two phenomena in this disease that make diagnosis even more difficult: genetic anticipation and incomplete penetrance, which add complexity to prognostic predictions (14–16). Due to this complexity, genetic testing must be part of the diagnostic evaluation of all pediatric patients with BMF, whether or not they present suggestive phenotypic manifestations (17). The objective of this Spanish multicenter study was to describe the natural course of DC in patients with long-term follow-up who were diagnosed in childhood and thus help to better understand the disease.

## Material and methods

Multicenter (4 Spanish centers provided the data of their patients), retrospective and longitudinal study of patients diagnosed with DC in childhood between 1998 and 2020 and followed up for 24 years.

Data were collected from the patients’ medical records after Clinical Research Ethics Committee approval. Ethical standards and legal requirements on the use of personal data were applied throughout the data collection stages.

### Definitions

***Mucocutaneous triad:*** anormal skin pigmentation, oral leukoplakia, and nails dystrophy (3, 5).

***Hematologic abnormalities:***

• Macrocytosis: mean corpuscular volume (MCV) of red blood cells >100 fl
• Macrocytic anemia: hemoglobin level <9 g/dl with MCV >100 fl
• Thrombocytopenia: platelet count <100 × 10^9^/L
• Neutropenia: neutrophil count <1.5 × 10^9^/L
• Pancytopenia: involvement of ≥2 hematopoietic lines
• Hypocellular bone marrow: overall bone marrow cellularity <30% as estimated by trephine biopsy.
• Bone marrow failure (BMF): peripheral pancytopenia associated with bone marrow hypocellularity as observed by trephine biopsy. Moderate: Not fulfilling criteria for severe or very severe. Severe: reticulocytes <20 × 10^9^/L, polymorphonuclear leukocytes (PMNs) 0.5–0.2 × 10^9^/L, platelets <20 × 10^9^/L. Very severe: PMNs <0.2 × 10^9^/L.

***Severe clinical variants:***

• Hoyeraal-Hreidarsson syndrome: characterized by intrauterine growth restriction, microcephaly, cerebellar hipoplasia (characteristic finding), early BMF, and variable immune deficiency (18, 19).
• Revesz syndrome: characterized by *bilateral exudative retinopathy (characteristic finding)*, nail dystrophy, BMF, fine hair, cerebellar hypoplasia, and growth restriction (20).

### Diagnosis

Diagnosis was established based on clinical criteria, telomere length in blood cells below the first percentile for age (4, 6, 13, 21) (quantitative PCR (qPCR) or more recently by flow fluorescence in-situ hybridization (2) (Flow-FISH)), and genetic tests (2, 12, 17) (next-generation sequencing of a panel of BMF genes, Sanger sequencing of selected exons or exome/genome sequencing). Since the patients belong to different eras, the panels of genes studied varied over time, expanding the number of genes studied as new genes were identified.

### Statistical analysis

Kaplan-Meier curves were used to estimate overall survival. Time to onset of specific features (mucocutaneous manifestations, bone marrow failure, pulmonary and liver fibrosis, or esophageal stricture) was based on cumulative incidence analysis with competing risks, death being the competitive event. Date of birth was considered the starting date for DC.

## Results

### Patient characteristics

Fourteen patients (eight boys and six girls) from 12 families were followed between 1998 and 2022 (Table 1).

**Table 1:** Associated mutation, clinical manifestations, treatments received and outcomes.

	1	2	3	4	5	6	7^a^	8^a^	9	10	11	12	13^a^	14^a^
Genetics finding														
Genetic mutation	*Double Heterozygous gen TINF2*	*Unidentified*	*Heterozygous gen TERT*	*Gen DKC1*	*Heterozygous gen RTEL1*	*Unidentified*	*Heterozygous gen TERT*	*Heterozygous gen TERT*	*Homozygous gen RTEL1*	*Double Heterozygous gen TINF2*	*Heterozygous gen TERT*	*Heterozygous gen TERT*	*Homozygous gen RTEL1*	*Homozygous gen RTEL1*
Inherence Pattern	*AR*	* *	*AD*	*AR*	*AD*	* *	*AD*	*AD*	*AR*	*AR*	*AD*	*AD*	*AR*	*AR*
Suggestive DC family history	* *	* *	* *	* *	* *	* *	*X*	*X*	* *	* *	* *	*X*	*X*	*X*
Clinical manifestations														
Severe clinical variants		SR	SHH	SHH					SHH	SHH			SHH	SHH
IUGR	X	X	X	X	X	X			X	X			X	X
Prematurity			X						X	X			X	
Microcephaly			X	X					X	X			X	X
Hematological involvement														
Macrocytosis	X	X	X	X	X	X	X	X	X	X	X	X	X	X
Platelets	X		X	X	X		X	X	X	X		X	X	X
Pancytopenia						X					X			
Classic triad														
Skin	X	X	X	X	X	X			X	X		X	X	
Mucous	X	X	X	X	X	X			X	X			X	
Nails	X	X	X	X	X	X								
Weight stagnation	X	X	X	X	X	X	X	X	X	X			X	X
Short size	X	X	X	X	X	X			X	X		X	X	X
Dental anomalies	X	X	X	X	X	X	X	X	X	X			X	
Musculoskeletal manifestations														
Osteoporosis	X	X	X	X	X	X	X	X	X	X		X	X	
Avascular bone necrosis	X	X		X	X				X	X		X	X	
Scoliosis			X									X		
Sparse hair/baldness		X	X	X	X	X			X					
Ophthalmological manifestations	X	X	X	X	X	X			X					
Neurological manifestations		X	X	X					X	X			X	X
Hepatic involvement														
Increased enzymes			X	X	X			X		X		X	X	
Steatosis												X		
Fibrosis		X	X	X	X			X						
PHT				X	X			X						
Esophageal stricture	X	X	X	X	X				X	X				
Pulmonary fibrosis			X	X	X									
Genitourinary manifestations	X		X	X	X				X					
Hormonal dysfunction			X	X	X							X		
Immunodeficiency			X										X	
Treatments received														
Blood product transfusions	X	X	X	X	X	X	X	X	X	X	X	X	X	
Androgens	X	X					X	X		X		X	X	
HSCT	X		X		X	X			X	X	X	X		
Clinical status														
Alive							X	X	X	X	X	X	X	X
Dead Cause of dead	Invasive fungal infection (*Aspergillus niger*)	Septic shock (dental/gum infection)	Septic shock (*Staphylococcus aureus*)	PF (respiratory failure)	Pneumonia (Morganella morganii)	TRM								

AD, autosomal dominant; AR, autosomal recessive; SV, revesz syndrome; SHH, hoyeraal-hreidarsson syndrome; IUGR, intrauterine growth retardation; Prematurity, children born before 37 weeks of gestation; PHT, portal hypertension; HSCT, hematopoietic stem cell transplantation; PF, pulmonar fibrosis; TRM, transplant-related mortality.

^a^ Pairs of siblings.

### Diagnosis

#### Family history

Five patients had a suggestive family history of DC: death of sibling due to unknown BMF (*n = *1), pulmonary fibrosis (*n = *2), acquired aplastic anemia (*n = *2), and clinical manifestations suggestive of Hoyeraal-Hreidarsson syndrome (*n = *2).

#### Initial diagnoses

Ten patients were initially diagnosed with a condition other than DC: immune thrombocytopenia (*n = *5), acquired aplastic anemia (*n = *4), and severe combined immunodeficiency (*n = *1).

#### Definitive diagnosis

A definitive diagnosis of DC was established at a median age of 8.5 years [range, 3–17 years]. Twelve patients (85%) had a telomere length below the first percentile, in 11 (91%) the diagnostic test used was qPCR and in 1 (9%) flow-FISH in peripheral blood nucleated cells. Telomere length was not measured in 2 patients. Mutations involving four genes were detected in 12 patients: *TERT* (*n = *5), *RTEL1* (*n = *3), *TINF2* (*n = *3), and *DKC1* (*n = *1). The inheritance pattern in patients with *TERT* mutations was AD in all cases, while, in patients with *RTEL* mutations it was AD in 1 and AR in 3. In the other two patients, the diagnosis was based on clinical findings (Table 1).

#### Family study

One or both parents had the same mutation as their child in 11 of the 12 families studied. No mutations were identified in the remaining set of parents. The sibling study detected two new cases of DC and one asymptomatic carrier.

### Hematologic abnormalities

**Figure 1:**
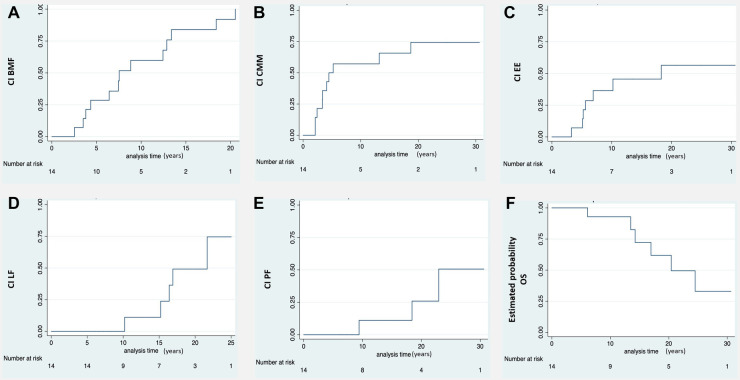
Ci of the different clinical manifestation of DC and estimated probability of OS. (**A**) Moderate-severe bone marrow failure (BMF). (**B**) Cutaneous-mucosal and phaneras manifestations (CMM). (**C**) Esophageal stricture (EE). (**D**) Liver fibrosis (LF). (**E**) Pulmonary fibrosis (PF). (**F**) Estimated probablility overall survival (OS). A progressive reduction in survival is observed due to the evolution of the disease and its complications during long-term follow-up. *CI, cumulative incidence. Number at risk: number of patients at risk of developing any DC complication.

The median age at onset of hematologic abnormalities was 3 years [range, 1 month-14 years]. In all patients an increase in fetal hemoglobin was identified, the mean value was 9% [range, 4%–19%]. The initial manifestations were macrocytosis (100%), thrombocytopenia (73%), and pancytopenia (21%). Ninety-two percent progressed to BMF with moderate to severe/very severe pancytopenia at a median age of 7 years [range, 3–20 years]. The bone marrow study showed hematopoietic hypocellularity in all cases. The likelihood of having moderate to severe/very severe BMF at the age of 10 and 20 years was 67% [CI 43%–87%] and 98% [CI 69%–99%] respectively (Figure 1A).

### Extra-hematological manifestations

At 10-year follow-up, all patients had developed 2 or more clinical manifestations, with the hematological phenotype being constant. Of the 6 patients who reached 20 years of follow-up, 5 of them developed 3 or more clinical phenotypes.

Intrauterine growth restriction resulting in a low birth weight and/or prematurity occurred in 71% of patients. The likelihood of developing mucocutaneous triad manifestations at 10 and 20 years was 65.7% [CI 41.3%–88%] and 75% [CI 49%–93.3%] respectively (Figure 1B). Eight patients developed nail defects and oral leukoplakia at a median age of 3.5 years [range, 2–13 years]. Seven patients experienced skin alterations (lacy reticulate pigmentation, hyper/hypopigmentation) at a median age of 6 years [range, 4–13 years]. Two developed only nail defects: one at 3 years of age and the other at 18.

All the patients had non-hematologic manifestations; the most common were weight stagnation and short stature, epiphora, musculoskeletal disorders (low bone mineral density, avascular necrosis of hip), dental abnormalities (caries, loss of teeth) and sparse hair or alopecia.

Seven patients (50%) were diagnosed with severe variants of DC: Hoyeraal-Hreidarsson syndrome in six cases and Revesz syndrome in one. The median age at diagnosis was 4 years [range, 3–13 years]. Patients with Hoyeraal-Hreidarsson Syndrome were born prematurely and had low birth weight. All of them presented with microcephaly, delayed psychomotor development, cerebellar hypoplasia with ataxia, dysmetria, and other extrapyramidal signs. The patient with Revesz syndrome had cerebral calcifications and exudative retinopathy that progressed to blindness; he died of septic shock secondary to a dental/gum infection at the age of 4 years.

Seven patients (50%) had esophageal stricture, which was symptomatic in five cases and asymptomatic in two. The probability of developing esophageal stricture at the age of 10 and 20 years was 45.5% [CI 23%–75%] and 56% [CI 31%–84%] respectively (Figure 1C). Stricture symptoms started at a median age of 6 years [range, 3–18 years], and all patients required dilatation.

Eight patients (57%) had liver involvement with elevated enzymes and variable alterations in echogenicity (*n* = 7), steatosis (*n* = 1), fibrosis (*n* = 5), and portal hypertension (*n* = 3). The probability of having developed liver fibrosis at the age of 10 and 20 years was 11.1% [CI 1.6%–56%] and 75% [CI 35.8%–98.5%] respectively (Figure 1D). The median age at diagnosis of portal hypertension was 14 years [range, 10–21 years].

**Figure 2:**
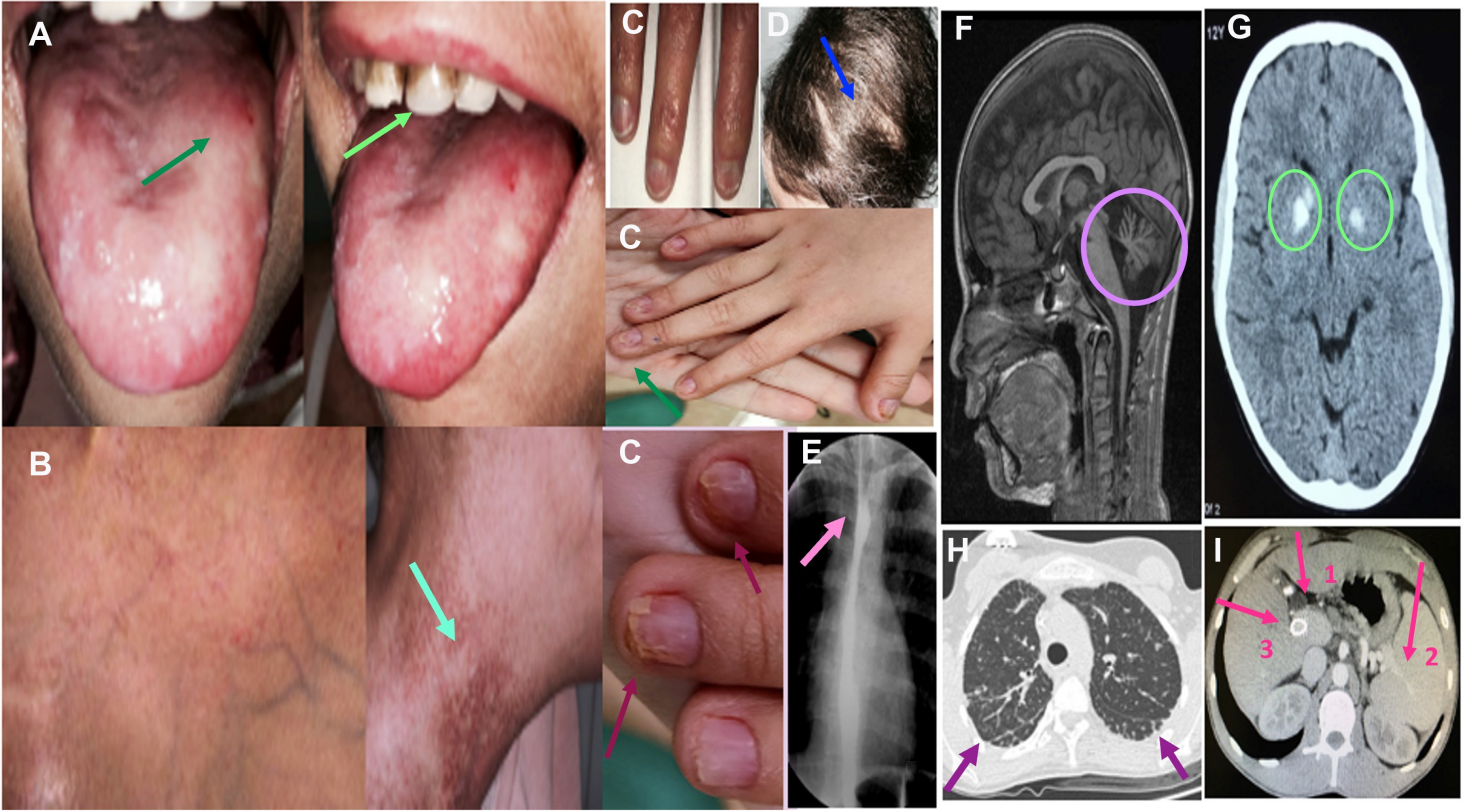
Clinical and radiological findings in DC. (**A**) Patient 5: oral leukoplakia and tooth involvement. (**B**) Patient 5: reticular pigmentation of the neck. (**C**) Patients 4, 9 and 10: nail dystrophy. (**D**) Patient 9: scarce and sparse hair. (**E**) Patient 9: Esophageal stricture. (**F**) Patient 3: Brain MR shows cerebellar hypoplasia in a patient diagnosed with Hoyeraal-Hreidarsson Syndrome. (**G**) Patient 2: Cranial CT shows calcifications of the basal ganglia in a patient with Revetz Syndrome. (**H**) Patient 3: Axial section of the chest CT showing signs of incipient pulmonary fibrosis with subpleural lines predominantly basal and heterogeneous distribution. (**I**) Patient 8: Abdominal CT showing signs of severe liver fibrosis: portal vein dilatation (arrow 1) and secondary splenomegaly (arrow 2). Transjugular intrahepatic portosystemic shunt (TIPS) is also shown (arrow 3).

Computed tomography of the chest showed evidence of pulmonary fibrosis in three patients (21%) with a median age of 17 years [range, 9–22 years]. Two were symptomatic (hypoxemia). The probability of having developed pulmonary fibrosis at the age of 10 and 20 years was 11.1% [CI 1.6%–56%] and 50% [CI 17%–92%] respectively (Figure 1E). Figure 2 shows the clinical and radiological findings of the extra-hematological manifestations of DC patients.

### Treatment

Thirteen patients received blood product transfusions. Seven were treated with androgens: two showed a transient response and two achieved transfusion independence. They all experienced adverse effects (dyslipidemia (*n* = 7), virilization (*n* = 2), metrorrhagia (*n* = 1), and liver dysfunction (*n* = 5)). Six received granulocyte colony-stimulating factor (G-CSF) but did not respond. Eight required a hematopoietic stem cell transplant (HSCT) due to very severe BMF at a median age of 6 years [range, 3–18 years]. Donors were mismatched family donor (*n* = 1), mismatched unrelated donor (*n* = 1) and matched unrelated donor (*n* = 6). The stem cell source was bone marrow (*n* = 6) and peripheral blood (*n* = 2). Reduced-intensity conditioning regimen was fludarabine-based combined with an alkylator (cyclophosphamide (*n* = 7) or busulfan (*n* = 1)). Low dose of total body irradiation (2 Gy) was added in transplants at high risk of graft failure (*n* = 6). GVHD prophylaxis consisted of cyclosporine plus mycophenolate mofetil (MMF) (*n* = 2) or cyclosporine plus methotetrexate (*n* = 6). All patients engrafted.

### Overall survival and clinical outcomes

Eight patients were still alive after a median follow-up of 9 years [range, 2–24 years]. The median age of these patients at the time of this study was 18 years [range, 6–32 years]. Four of the eight patients underwent HSCT and achieved normal blood counts and full donor chimerism. The non-HSCT patients (*n* = 4) experienced progression of BMF: three developed moderate-severe BMF, and one had isolated low platelet counts. None of the eight patients had developed myelodysplastic syndrome or cancer by the time of the study.

The median age of death in the six patients who died was 13 years [range, 6–24 years]. Four had undergone HSCT. One died on day +60 due to transplant-related toxicity and three died more than 2 years later due to infections: an invasive fungal infection (*Aspergillus niger*), septic shock (*Staphylococcus aureus*), and pneumonia (*Morganella morganii*). The two patients who did not undergo HSCT died of septic shock secondary to a dental/gum infection in one case and respiratory failure due to pulmonary fibrosis in the other.

## Discussion

DC is an ultra-rare hereditary disease with a variable clinical presentation and high morbidity and mortality (2, 4, 6, 22). This study provides information on the natural course of the disease through the study of a small cohort of Spanish patients diagnosed in childhood and followed up for a long period of time, which has allowed to observe the appearance of several extra-hematological complications. Our findings are consistent with largest published series (22). DC appears in childhood in most cases as a multisystemic disease, and its hallmark signs are the mucocutaneous triad and hematologic manifestations (4, 6, 23). Diagnosis is a challenge since many patients are initially diagnosed with other clinical conditions such as immune thrombocytopenia (ITP), immunodeficiency or others (16). In our series this happened in 71% of patients.

Sixty-four percent of patients had mucocutaneous triad from an early age. Nail defects and oral leukoplasia were more common than skin manifestations and appeared earlier, consistent with other reports (2, 4, 6). All the patients developed hematologic alterations at a very young age, and 64% progressed to severe BMF in the first decade of life, in our cohort the BMF was earlier than in other reports (usually 20 years) (2, 4, 6). Half of the patients had a severe phenotype, with multisystemic involvement and an early diagnosis, reflecting the early onset of hematological and extra-hematological manifestations in children affected by these variants.

Liver and lung involvement in the first two decades of life was more common than reported previously (1, 2, 4, 6, 23). Liver failure and portal hypertension, for example, were five and three times more common, respectively (1, 2, 4, 6, 23).

*DKC1* has been identified in 20%–30% of DC patients in the literature (1, 2), making it the most common gene in this setting (1, 2, 23), In our series, however, it was detected in just one patient. The most common mutations in our group were in *TERT* (35%), *RTEL1* (21%), and *TINF2* (21%). The frequencies reported in other series for these mutations are variable being 7%–23%, 7%–14%, and 12% respectively (1, 2, 23).

Thirteen of the fourteen patients in our series developed moderate to severe BMF during follow-up. BMF is one of the most common and lethal conditions in DC (1, 2, 16, 23). Seven patients were treated with androgens. Androgen therapy can improve hematopoietic function for variable periods of time, although the mechanisms are poorly understood (24, 25). Four of the patients (57%) responded initially to androgens, supporting previous reports (60%–70%) (24, 25), but just two (28%) maintained this response. Androgens should be avoided in neutropenic patients treated with G-CSF due to the risk of splenic peliosis and rupture (26). HSCT is the only potentially curative treatment for BMF. It is associated with overall survival rates of 66% at 3 years and 55% at 5 years (27). The best outcomes have been observed in patients younger than 20 years without multiorgan involvement, the use of reduced-intensity conditioning regimen based on fludarabine, HLA-matching, and bone marrow cells as graft source (27, 28). HSCT survival rates in our series are like those reported elsewhere (27–29).

Although mortality after HSCT is not negligible in DC, most deaths are caused by disease- rather than transplant-related complications, such as infections, pulmonary and hematological complications, and cancer (27–29). Similar findings were observed in our series. Given the risk of HSCT-related complications, the multicenter prospective trial (NCT01659606) evaluating engraftment after a radiation and alkylator free-conditioning regimen is promising, as removal of DNA-damaging agents may reduce the risk of HSCT-related complications and allow transplantation in patients with high-risk comorbidities (30).

Multidisciplinary follow-up, together with cancer prevention and early detection strategies, is important in long-term survivors of DC due to the increased risk of hematologic (myelodysplastic syndrome/acute myeloid leukemia) and non-hematologic cancer (squamous cell carcinoma of head and neck and anogenital region) after the age of 30 years (31–33). Alter et al, report a high incidence of squamous cell carcinoma of the tongue, mainly in patients with a history of leukoplakia (pre-malignant lesion). Therefore, regular examination and proper care of the oral cavity is essential (31). At the time of the study, none of the patients in our series had developed cancer, but the oldest patient was just 32 years old.

Long-term follow-up findings in this series shows that hematologic manifestations appear early and are very common, that non-hematologic manifestations are progressive, and that morbidity and mortality rates are high. However, the study does have certain limitations, on one hand, the small number of patients is a result of the low prevalence of DC and the absence of an official Spanish Registry. On the other hand, the early diagnosis in childhood primarily identifies severe phenotypes, while excluding late oligosymptomatic or monosymptomatic presentations that may occur in adults.

Adequate transition from pediatric to adult care is essential. Given the absence of specific clinical guidelines in Spain, it is encouraged to develop such guidelines to ensure optimal multidisciplinary follow-up, implement cancer prevention measures and genetic counselling.

## Data Availability

The original contributions presented in the study are included in the article/Supplementary Material, further inquiries can be directed to the corresponding author.
